# Coastal Microbial Mat Diversity along a Natural Salinity Gradient

**DOI:** 10.1371/journal.pone.0063166

**Published:** 2013-05-21

**Authors:** Henk Bolhuis, Lucas Fillinger, Lucas J. Stal

**Affiliations:** Department of Marine Microbiology, Royal Netherlands Institute of Sea Research (NIOZ), Yerseke, The Netherlands; Argonne National Laboratory, United States of America

## Abstract

The North Sea coast of the Dutch barrier island of Schiermonnikoog is covered by microbial mats that initiate a succession of plant communities that eventually results in the development of a densely vegetated salt marsh. The North Sea beach has a natural elevation running from the low water mark to the dunes resulting in gradients of environmental factors perpendicular to the beach. These gradients are due to the input of seawater at the low water mark and of freshwater from upwelling groundwater at the dunes and rainfall. The result is a natural and dynamic salinity gradient depending on the tide, rainfall and wind. We studied the microbial community composition in thirty three samples taken every ten meters along this natural salinity gradient by using denaturing gradient gel electrophoresis (DGGE) of rRNA gene fragments. We looked at representatives from each Domain of life (Bacteria, Archaea and Eukarya) and with a particular emphasis on Cyanobacteria. Analysis of the DGGE fingerprints together with pigment composition revealed three distinct microbial mat communities, a marine community dominated by diatoms as primary producers, an intermediate brackish community dominated by Cyanobacteria as primary producers and a freshwater community with Cyanobacteria and freshwater green algae.

## Introduction

More than fifty percent of the North Sea beach of the Dutch barrier island Schiermonnikoog is covered by a vegetation rich salt marsh [1]. The development of this salt marsh is the result of the establishment of coastal microbial mats that stabilized the sediment, increased the erosion threshold and enriched the sediment with organic matter and nutrients [2], [3]. Hence, coastal microbial mats are important mediators of natural coastal protection and morphodynamics.

Coastal microbial mats are considered as the modern analogues of fossil stromatolites, the oldest of which date back almost 3.5 billion years and represent the oldest ecosystems known [4].The stratified mats consist of three main layers [2], [3]. The top green layer is dominated by primary producers (mainly Cyanobacteria and diatoms) that fix carbon dioxide through oxygenic photosynthesis enriching the sediment with organic carbon. Some Cyanobacteria are capable of fixing di-nitrogen (N_2_) which makes them independent on a source of combined nitrogen while providing a source of nitrogen for the whole microbial community. A distinct purple layer is often visible directly below the green layer and consists of anoxygenic phototrophic purple sulfur bacteria. Their substrate, sulfide, is produced by anaerobic sulfate reducing bacteria (SRB). The SRB do not occur in a distinct layer and some species that can tolerate or even respire oxygen can be found near the surface where labile organic carbon is available [5]–[7]. Their presence in the lower layers can be deduced from the black horizon of iron sulfide, in the permanently anoxic layer below the purple sulfur bacteria. Microbial mats contain a variety of different functional groups of microorganisms of which their location do not necessarily follow the vertically stratified layers. These microorganisms include autotrophic colorless sulfur bacteria, photoheterotrophic non-sulfur bacteria, a variety of chemotrophic Bacteria and Archaea as well as protists (fungi, algae and nematodes) and insects [8]. Previous studies indicated the presence of 3 to 5 different types of microbial mats with dimensions of approximately 100 meters wide and stretching several kilometers over the northwest part of the North Sea coast of Schiermonnikoog [1], [9]. An extensive analysis of the archaeal and bacterial diversity in these mats by massive parallel 16S rRNA tag sequencing revealed that these apparent simple layered structures harbor one of the most diverse marine microbial ecosystems with a plethora of different functional groups of Bacteria and Archaea [10]. A single sample was analyzed from three mat types that appeared to be different in terms of morphology and microscopic composition. These three stations were roughly characterized as a marine station in the tidal zone, an intermediate, brackish station, and a station in the supralittoral zone near the dunes that predominantly receives freshwater. The bacterial and archaeal community composition depended on the location and the type of mat rather than on the season. It was concluded that the community composition and the microbial diversity were intrinsic of the mat type and depended on the location along the tidal salinity gradient. However, this study did not take into account spatial heterogeneity of the microbial community. Large variations in the microbial community composition may occur within short distances and are considered the result of very different environmental conditions that produce its own distinct “micro-landscape” [11]. Distribution of species over distinct ecological zones and their boarders are well studied in vegetation ecology (e.g. [12]). Two main theories were proposed to describe distribution of plants along environmental gradients. The first, the continuum concept, suggests that each species has its own environmental tolerance and responds individualistically to the environmental gradient with their boundaries determined by their tolerances to environmental variables [13]. Alternatively, Clements [14] proposed a more holistic view with a tight linkage and cooperation among species for the benefit of the community. The current agreement is that species are distributed individualistically and that community composition typically changes along environmental gradients. Abrupt changes can be found but are often associated with abrupt changes in environmental parameters. With respect to the boarders different patterns are also recognized. These are known as *limes convergens* when a sharp boundary exists between communities resulting from an abrupt change in one or more environmental variables leading to the convergence or coincidence of population boundaries [15]. *Limes divergens* occurs when there is a gradual change in environmental conditions and populations gradually merge into one another without clear community boundaries.

This investigation aimed at providing a better analysis of the spatial diversity of the different mat types. A transect of the beach was sampled at ten meter intervals, ranging from the low water mark to the dunes. This transect included the stations that were sampled for the tag-sequencing reported in [10]. In addition to the bacterial and archaeal community, micro-eukaryotes were included in this study and a specific emphasis was put on Cyanobacteria. Rather than repeating an extensive rRNA gene sequencing program for many samples we compared microbial communities by the rapid, high resolution fingerprinting method based on denaturing gradient gel electrophoresis (DGGE) [16]. Cluster analysis of the community fingerprints was made in order to compare and group the fingerprints according to their similarity. The outcome of these experiments is discussed in the light of the theory on population changes and boundaries used in vegetation ecology.

## Materials and Methods

### Sample Site and Sampling

**Figure 1:**
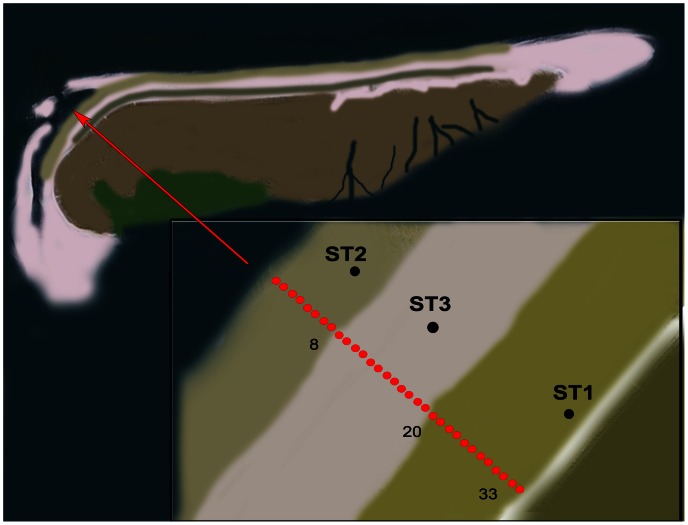
Sketch of geographical location of the sampling stations (red dots in the insert) at the north-western part of the North Sea coast of the Dutch barrier island Schiermonnikoog. The stations indicated with ST1, ST2 and ST3 refer to the stations sampled in a previous study [10] to which the current dataset is compared. See Materials and Methods for a description of the stations.

Samples were taken on August 25^th^, 2010 from microbial mats developing on the North Sea beaches of the Dutch barrier island Schiermonnikoog (Figure 1). Samples were taken at approximately 10 m intervals starting from the low water mark towards the dunes. Samples of 10–12 cm^3^ of the upper 5 mm of the microbial mat were collected in sterile 15 ml plastic screw cap tubes and stored at −20°C until analysis. In total 33 samples were taken (Transect Sample TS01-33).

### Nucleic Acid Extraction

Total community DNA was isolated from 250 mg (wet weight) of sediment using the MO-BIO UltraClean Soil DNA Isolation Kit (MO BIO Laboratories, Inc., Carlsbad, CA, USA) according to the manufacturer’s protocol for maximum yields. The concentrations of DNA were measured spectrophotometrically using the NanoDrop™ ND-1000 spectrophotometer (NanoDrop products, Wilmington, DE, USA). For controlled and reproducible downstream analysis the DNA concentration for each sample was adjusted to 6 ng/µl by adding diethylpyrocarbonate treated water.

### Ribosomal RNA Gene Amplification

Different polymerase chain reactions (PCR) were performed to amplify rRNA gene fragments from the Bacteria, Archaea, Eukarya and Cyanobacteria. The primers used are listed in Table 1. Archaeal, cyanobacterial and eukaryotic gene fragments were amplified in a nested PCR reaction since direct amplification with the DGGE primers were unsuccessful or delivered insufficient amounts of product. In the first PCR, part of the 16S or 18S rRNA gene was amplified using standard primers. The amplicons served as template for the second PCR where the dedicated DGGE primers were used. Reactions and PCR conditions were optimized for each primer pair and if applicable for each step of a nested PRC reaction. This resulted in the use of different polymerases and different concentrations of BSA, DMSO and *Taq* DNA polymerase. The reaction mixture composition and reaction conditions are listed in Table 2. PCR was carried out using a thermal cycler (Thermal Cycler 2720, Applied Biosystems, Foster City, CA, USA).

**Table 1:** List of oligonucleotides used in this study.

Primer Name	Specificity	Sequence, 5'–3'	Reference
F968-GC	Bacterial	**CGCCCGGGGCGCGCCCCGGGCGGGGCGGGGGCACGGGGGG** CCTACGGGAGGCAGCAG	[46]
1401R	Bacterial	CGGTGTGTACAAGACCC	[46]
U1492R	prokaryote	GGTTACCTTGTTACGACTT	[47]
CYA359f-GC	Cyanobacterial	**CGCCCGCCGCGCCCCGCGCCGGTCCCGCCGCCCCCGCCCGG**GGGAATYTTCCGCAATGGG	[48]
CYA781Ra	Cyanobacterial	GACTACTGGGGTATCTAATCCCATT	[48]
CYA781Rb	Cyanobacterial	GACTACAGGGGTATCTAATCCCTTT	[48]
A2F	Archaeal	TTCCGGTTGATCCYGCCGGA	[47]
SAF341F	Archaeal	CTAYGGGGCGCAGCAGG	[49]
PARCH519R-GC	Archaeal	**CGCCCGCCGCGCGCGGCGGGCGGGGCGGGGGCACGGGGGG**TTACCGCGGCKGCTG	[50]
EK1F	Eukaryote	CTGGTTGATCCTGCCAG	[51]
EK1520R	Eukaryote	CYGCAGGTTCACCTAC	[51]
EUK516r-GC	Eukaryote	**CGCCCGGGGCGCGCCCCGGGCGGGGCGGGGGCACGGGGGG** ACCAGACTTGCCCTCC	[52]

*Y = C or T, K = G or T.*

**Table 2:** Polymerase chain reaction mixtures and amplification conditions.

	Bacteria	Archaea		Eukarya		Cyanobacteria	
	16S-DGGE	16S	16S-DGGE	18S	18S-DGGE	Cyano-16S	Cyano -DGGE
Reaction type	Touch down^1^	Nested 1	Nested 2 touch down	Nested 1	Nested 2	Nested 1	Nested 2 touch down
1× PCR reaction mix	GE	HS	GE	HS	GE	HS	GE
BSA	–	0.01% w/v	–	0.01% w/v	0.01% w/v	0.01% w/v	–
DMSO	3% v/v	5% v/v	3% v/v	5% v/v	3% v/v	5% v/v	3% v/v
dNTPs	200 µM	200 µM	200 µM	200 µM	200 µM	200 µM	200 µM
Forward primer	F968-GC	A2F	SAF41F	EK1F	EK1F	CYA359f	CYA359f-GC
Reverse primer	U1492R	U1492R	PARCH519GC	EK1520R	EUK516r-GC	U1492R	CYA781Ra/b^2^
*Taq* polymerase^5^	1 U/µl (GE)	2 U/µl (HS)	1 U/µl (GE)	2 U/µl (HS)	1 U/µl (GE)	2 U/µl (HS)	1 U/µl (GE)
Initial denaturation	3′ at 95°C	15′ at 94°C	3′ at 95°C	15′ at 94°C	3′ at 95°C	15′ at 94°C	3′ at 95°C
# cycles 1st part	10	35	10	35	35	35	10
Denaturation	60″ at 95°C	60″ at 94°C	60″ at 95°C	60″ at 94°C	30″ at 95°C	60″ at 94°C	60″ at 95°C
Annealing	60″–60°C/55°C^4^	30″at 54°C	60″at 60°C/55°C^4^	30″at 54°C	45″ at 56°C	30″at 54°C	60″–60°C/55°C^4^
Extension	120” at 72°C	120” at 72°C	120” at 72°C	90″ at 72°C	130” at 72°C	90″ at 72°C	120” at 72°C
Final extension^3^	–	7′at 72°C	–	7′at 72°C	30′ at 72°C	7′at 72°C.	–
# cycles 2^nd^ part	25	–	25	–	–	–	25
Denaturation	60″ at 95°C	–	60″ at 94°C	–	–	–	60″ at 95°C
Annealing	60″ at 56°C	–	60″at 56°C	–	–	–	60″ at 56°C
Extension	120” at 72°C	–	120” at 72°C	–	–	–	120” at 72°C
		–		–	–	–	
Final extension^3^	30′ at 72°C	–	30′at 72°C	–	–	–	30′ at 72°C

1 Symbols used: “ = seconds, ‘ = minutes.

2 Two separate reactions were carried out with 2 different reverse primers (CYA781Ra & CYA781Rb) according to [48]. After amplification, samples were pooled, mixed and loaded on the DGGE gel.

3 A final extension of 30 minutes at 95°C was applied in order to prevent band duplication according to [53].

4 en step touch down with annealing temperature decreasing −0.5°C per cycle.

5 Polymerases used: GE = GE Healthcare *Taq* polymerase, HS = Qiagen HotStar *Taq* polymerase.

### Denaturing Gradient Gel Electrophoresis

DGGE-PCR reaction mixtures were purified using E.Z.N.A. CyclePure columns (Omega Bio-Tek, Inc., Norcross, GA, USA). After purification, the DNA concentration in each sample was determined spectrophotometrically and adjusted to a final concentration of 200 ng of DNA in 28 µl, supplemented with 2 µl of loading buffer [17] and applied on the DGGE gel. The DGGE gel was run on the Ingeny phorU® system (Ingeny International, Goes, The Netherlands). Denaturing polyacrylamide gels (8% w/v acrylamide) were made according to the manufacturer’s instructions using the gradient maker provided by the manufacturer in order to generate a urea-formamide gradient. The gradients ranged from 50–70% v/v for bacterial and cyanobacterial amplicons, 40–70% v/v for archaeal amplicons and 20–50% v/v for eukaryote amplicons. To 24 ml of the acrylamide-urea-formamide solution 50 µl 20% w/v ammonium persulfate and 5 µl of tetramethylethylenediamine were added to initiate polymerization. The stacking gel consisting of 8% w/v acrylamide but lacking the denaturants was poured on top of the gel. Samples and reference samples were subjected to electrophoresis at 100 V for 18 h in a 0.5 strength TEA buffer at 60°C. Reference samples were made by combining samples that covered the full gel gradient and re-amplifying these to generate larger quantities. After electrophoresis, DGGE gels were silver stained using an automated gel stainer (Hoefer Processor Plus, Amersham Biosciences). The following staining protocol was used: gel fixation was achieved by soaking the gel for 30 min in a solution of 0.05% v/v acetic acid and 10% v/v ethanol. Gel staining was performed in a 0.2% w/v silver nitrate solution for 15 min, followed by three 1 min washing steps with Milli-Q water. After washing, the gels were processed for 5 min with a developing solution consisting of 1.5% w/v sodium hydroxide and 0.15% v/v formaldehyde. Finally, gels were soaked in 0.75% w/v sodium carbonate for 5 min to stop the development and subsequently conserved by adding 10% v/v glycerin in 25% v/v ethanol and incubated for 7 min. For each step, 200 ml of the solutions was used.

### Community Fingerprint Analysis

The DGGE fingerprints were analyzed using the BioNumerics software package (Applied Maths NV, Sint-Martens-Latem, Belgium). Bands were automatically assigned and checked for false bands generated by silver stain spots in the gel. Using the DICE/Jackard band matching algorithm, the software calculated similarities between banding patterns in each sample lane and plotted the values in a similarity dendrogram. Some branches were swapped using the Bionumerics software to stress the observed conservation of sampling position within the cluster analysis. This only affected the visual presentation but left the clustering intact.

For microbial diversity analysis we designated distinguishable bands in the gel as an operational taxonomic unit (OTU). Bands occurring at the same height in different samples were considered to belong to the same OTU whereas their intensities were taken as measure of their relative abundance in the original sample. OTU position and intensity data were exported to the open-source statistical software package PAST [18]and used to calculate the Shannon diversity index [19] for each DGGE pattern. Redundancy analysis on environmental and DGGE data was performed using Canoco for Windows 4.5 [20].

### Physical Parameters

Water content was measured by weighing samples before and after freeze-drying. The salinity could not be measured directly due to limited amounts of pore-water. The relative salinity was indirectly determined by weighing 200 mg of freeze-dried sediment, adding 200 µl of ultra-pure Milli-Q water, vigorously vortexing for one minute and equilibrating for one hour. Salinities were then determined using a refractometry and corrected for the water content.

### Pigment and Nutrient Analysis

Pigments were extracted from freeze-dried sediment in 90% acetone and analyzed by HPLC (Waters Millennium HPLC system) using a reversed-phase analytical column (Novapak C18 column) [21]. Pigments were identified and quantified at 450 nm by comparison of retention times and photodiode array detection absorption spectra with a library of standards. For total carbon, nitrogen and phosphorus analysis, lyophilized samples were grinded and combusted with an excess oxygen at 1010°C. Nitrous oxides were reduced to N_2_ on a copper column at 650°C. CO_2_ and N_2_ gases were separated and quantitated using a Fisons NA-2500 elemental analyzer on a GC column (Haysep-Q) and with a thermal conductivity detector. Phosphorus compounds were measured colorimetrically on a SEAL quAAtro high performance microflow analyzer (Bran+Luebbe, Norderstedt, Germany).

In order to compare the relative distribution of the different pigments and nutrients along the beach transect, the values at each sample were normalized by the chlorophyll *a* (chl-*a*) concentration in that sample. The relative contribution of pigments to a particular zone was calculated by summing the percent contribution of samples grouped in that zone in which the sum of all concentrations for each pigment was set at 100%. One way ANOVA tests (p<0.05) were performed in combination with Tukey’s Post Hoc test using PAST [18] on all data sets to establish significance of observed differences.

## Results

### Sampling

The thirty three samples collected from the North Sea beach of Schiermonnikoog covered a nearly full transect of the beach which can be separated in three zones. The tidal zone is situated between the low- and high water mark and is inundated twice a day for up to 6 h throughout the year. The microbial mats in this zone have a gelatinous, sandy appearance and diatoms are an important component of the surface of the mats. The intermediate zone is inundated only during spring tide that occurs approximately one week per month and consisted of well developed, rigid microbial mat structures that were previously identified as mats dominated by filamentous Cyanobacteria such as *Coleofasciculus* sp. (previously known as *Microcoleus* sp. [22]) and *Lyngbya* sp. [1], [9], [10]. The transition to the upper zone is less clear. This zone is located closer to the dunes, is largely overgrown by vegetation and is mainly exposed to freshwater from rainfall and upwelling groundwater. Seawater only reaches this zone a few times per year at spring tides in combination with strong northerly winds. In total, the sampled transect covered nearly 350 meters of the beach. The sampling points overlapped with previously sampled stations for which we obtained an in depth analysis of the microbial composition [10].

### DGGE Community Fingerprinting

**Figure 2:**
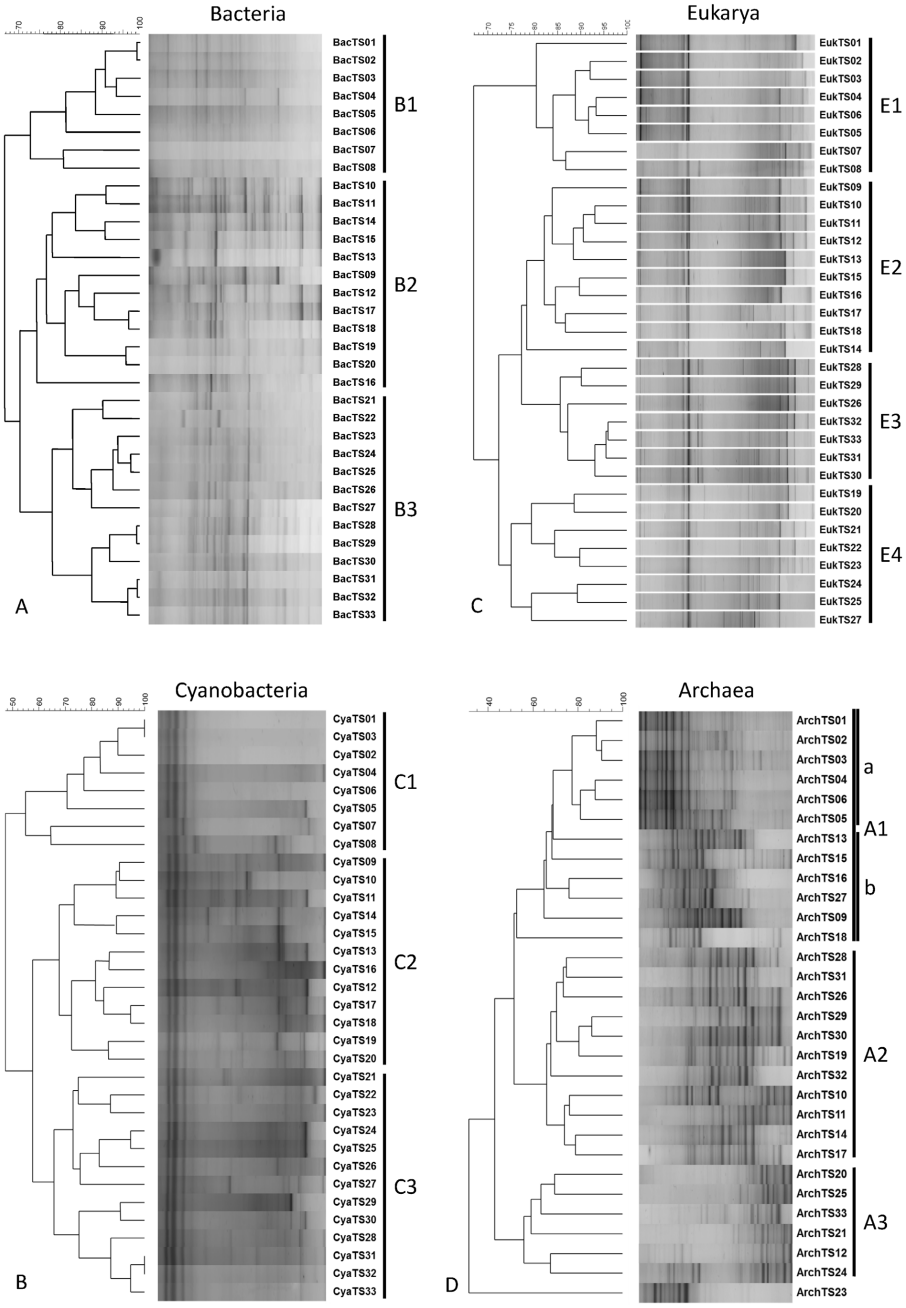
Cluster analysis of microbial community fingerprints and concomitant DGGE patterns for a) bacterial community, b) cyanobacterial community, c) eukaryal community and d) archaeal community.

Cluster analysis of bacterial community fingerprints obtained by DGGE analysis revealed three main clusters, designated B1, B2 and B3 (Figure 2a). The sampling position is strongly conserved within the clusters. Cluster B1 consisted of samples TS1 through TS8, cluster B2 of samples TS9 through TS20 and cluster B3 of samples TS21 through TS33. Furthermore, Figure 2a shows that cluster B2 and B3 have more bands in common than each of them with cluster B1. None of the fingerprints within a cluster was 100% identical suggesting variations in community composition between adjacent samples. Neighboring samples TS8 and TS9, which clustered in a different group, revealed large differences in composition. The cyanobacterial DGGE fingerprint followed the same clustering as was found for the total bacterial community and revealed three distinct clusters (C1–C3) (Fig. 2b). Clustering of the eukaryal DGGE fingerprints revealed 4 major clusters (E1–E4) of which only the first, E1, overlapped with the first bacterial and cyanobacterial cluster and thus consists of the same 8 samples (TS1-TS8) (Figure 2c). Cluster E2 is formed by the fingerprints of samples TS9-TS18, cluster E3 by fingerprints of the samples TS19-TS25 and TS27 and cluster E4 consists of the fingerprints of samples TS28-S33 plus TS26. The archaeal community fingerprints formed three clusters (A1-A3) (Figure 2d). Samples TS1-TS6 (Samples TS7 and TS8 are lacking because of insufficient archaeal DGGE products for further analysis) are highly similar and form a well-defined sub-cluster within A1. The other fingerprints within cluster A1 are less similar to each other. Two additional clusters A2 and A3 were identified but revealed low conservation with respect to the sampling position whereas sample TS23 appeared as a single branch. Based on the bacterial, cyanobacterial and eukaryal DGGE fingerprints we decided to combine the samples in 3 groups representing the tidal zone (TS1-TS8), the intermediate zone (TS9-TS20) and the upper zone (TS21-TS33), respectively. This zone division is used below to further characterize the beach transect.

### Diversity Analysis

**Figure 3:**
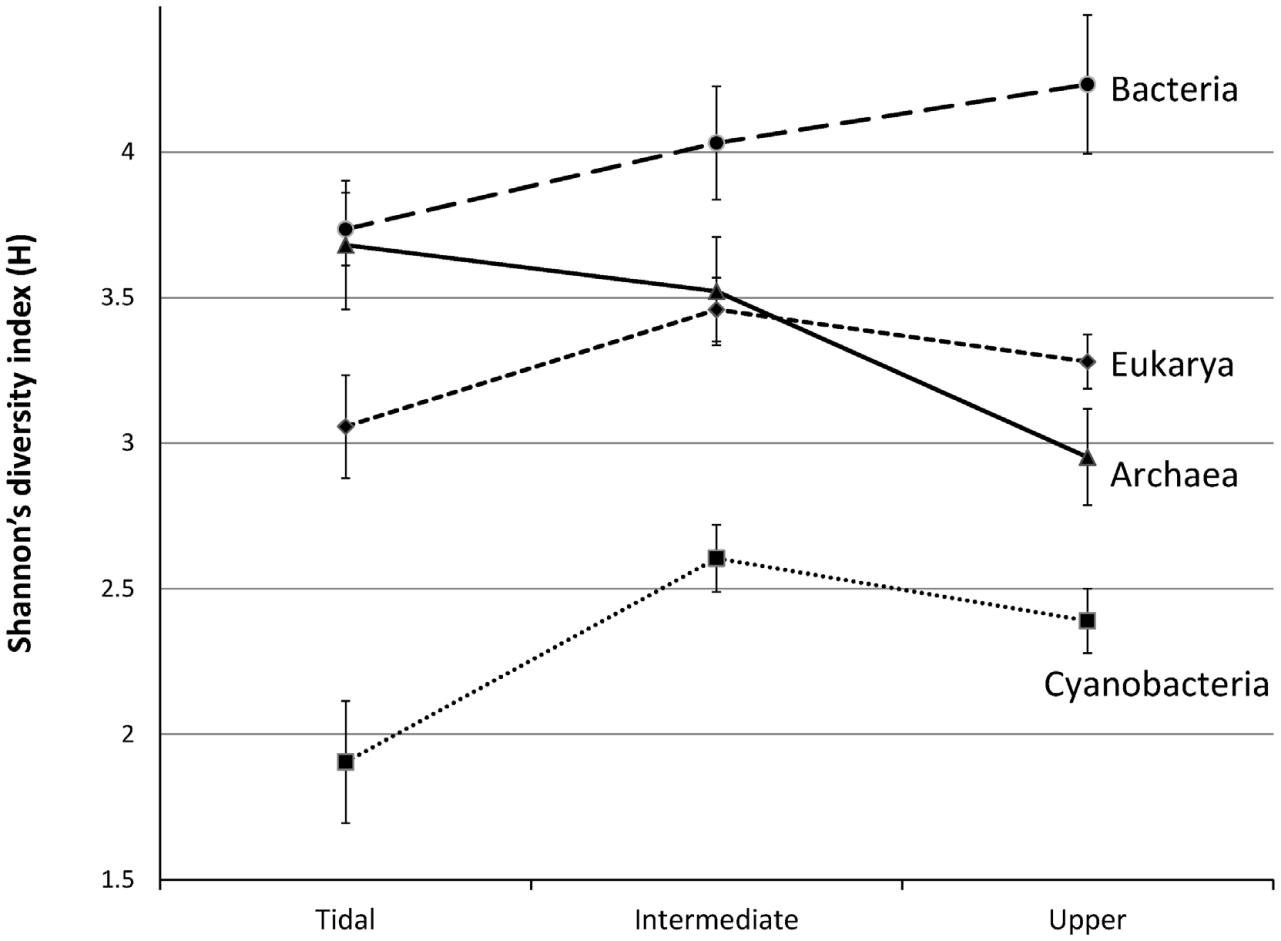
Shannon diversity index (h) of the four different microbial communities averaged per zone.

Transformation of the densitometric information to a data-array allowed us to perform a community diversity analysis in which band-position and band-intensity represented the operational taxonomic units and their relative abundance, respectively. The calculated Shannon diversity index varied per sampling point and gave an average index of 3.29+/−0.43 for Bacteria, 2.35+/−0.35 for Cyanobacteria, 3.33+/−0.43 for Archaea and 4.04+/−0.40 for Eukarya. The average diversity index per zone was also calculated as deduced from the DGGE cluster analysis (Figure 3). ANOVA and Tukey’s Post hoc test were performed to test the significance of the averages. The bacterial and cyanobacterial diversity were significantly lower in the tidal zone compared to the intermediate and upper zone. The eukaryal diversity was also lowest in the tidal zone while the intermediate and upper zone did not significantly differ from each other. Only the archaeal fraction followed an opposite trend with a significant lower diversity in the upper zone.

### Distribution of Biotic and Abiotic Factors

The concentration of the various pigments and nutrients fluctuated strongly between the different samples. The total amount of chlorophyll *a* in the samples TS1-TS8 was significantly lower compared to the other zones with an average concentration of 18.3 µg chl-*a*/gr dry-weight versus 190.6 µg chl-*a*/gr dry-weight in the intermediate zone and 67.5 µg chl-*a*/gr dry-weight in the upper zone. The concentrations in the samples from the two higher zones varied widely with the highest concentration found in sample TS9 (>600 µg chl-*a*/gr dry-weight). For further pigment and nutrient analysis and comparison we used their relative amounts, i.e. the amount of pigment normalized to the amount of chl-*a* in that sample.

**Figure 4:**
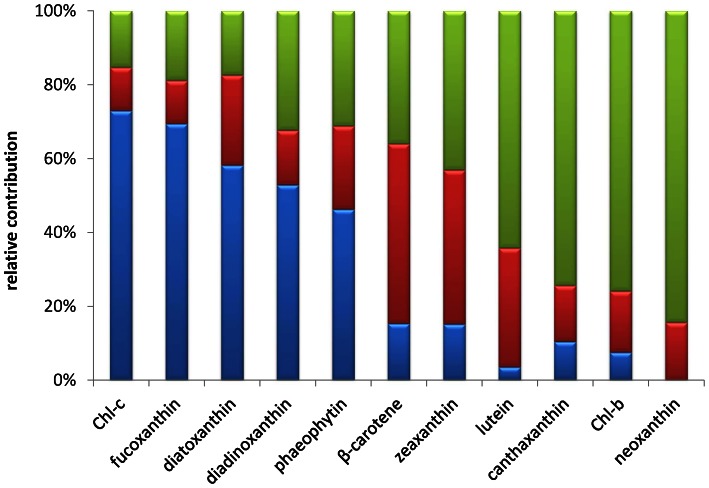
Relative contribution of photosynthetic pigments to the tidal (blue), intermediate (red) and upper (green).

A succession of different dominating pigments along the beach transect was observed (Figure 4). Chlorophyll-*c* and fucoxanthin were most abundant in the tidal zone whereas beta-carotene was highest in the intermediate and upper zone. Compared to the other zones, the tidal zone was also rich in diadinoxanthin, diatoxanthin and phaeophytin. Zeaxanthin content was highest in the intermediate and upper zone. Lutein, canthaxanthin, chlorophyll-*b* and neoxanthin were present in relative low quantities compared to the other pigments and mainly found in the upper zone.

**Figure 5:**
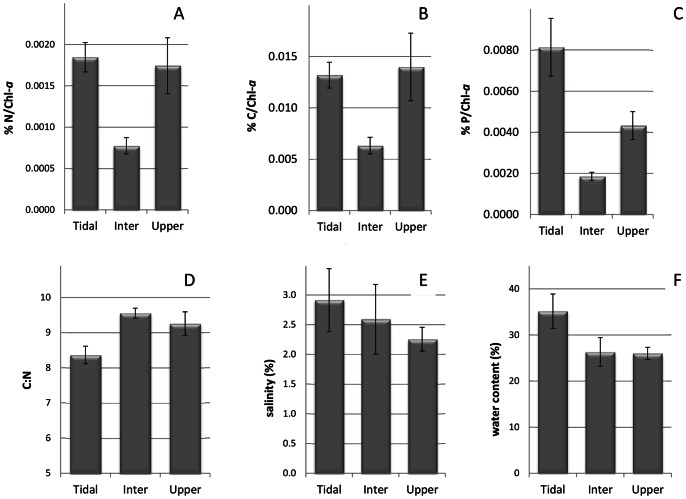
Average values per zone of total carbon (a), nitrogen (b), phosphate (c), C:N ratio (d), salinity (e) and water content (f). Carbon, nitrogen en phosphate concentrations (in % w/w) of each sample were normalized for the chlorophyll-*a* concentration in that sample before averaging.

Both nitrogen and carbon content were almost 2 fold lower in the intermediate zone than in the other two zones (Fig. 5a & b). The phosphate concentration was lowest in the intermediate zone and significant higher in the tidal zone (Figure 5c). Overall the C:N ratio along the gradient varied from 6.8 to 10.9. Both the minimum and the maximum C:N values were found in samples derived from the upper zone. The average C:N ratio per zone (Figure 5d) was lowest in the tidal zone (8.36+/−0.70) while the intermediate and upper zone had similar values (9.55+/−0.45 and 9.26+/−1.2 respectively). Water content (Figure 5e) and salinity (Figure 5f) were highest in the tidal zone and decreased towards the upper zone.

### Ordination Analysis

**Figure 6:**
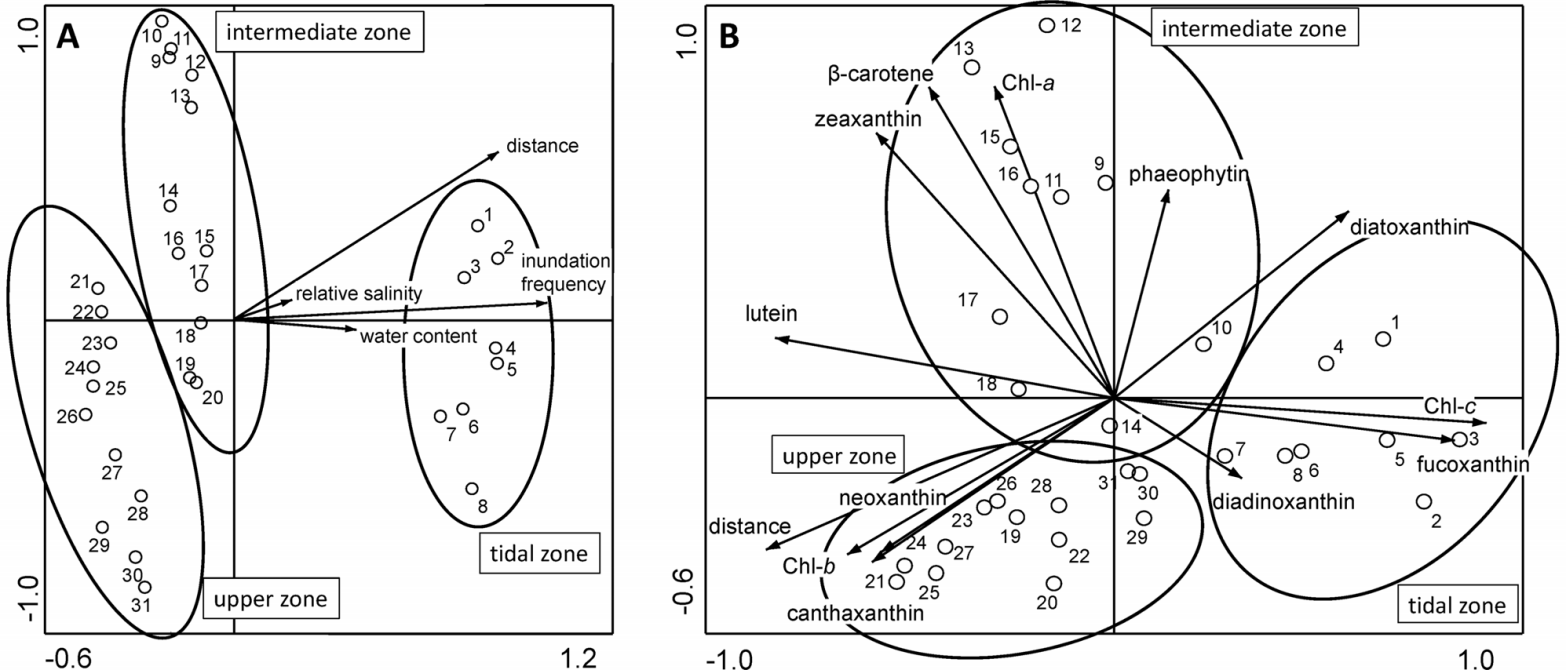
RDA plot of bacterial community DGGE profiles of the transect samples along with the most significant environmental parameters (A) and pigments (B). The ovals around the groups indicated the independent clusters of samples from the different mat types. Eigenvalues for axes 1 and 2 in A are 0.1611 and 0.1163, respectively, accounting for 28% of the total variance and in B are 0.1795 and 0.1082 respectively, accounting for 29% of the variance in the DGGE pattern data.

Redundancy ordination analysis (RDA) was performed to determine which environmental factors were the most significant to explain variation in the bacterial community composition. All four environmental parameters, salinity, water content, distance from the low water mark and inundation frequency point to the same direction with the latter two having the most significant contribution (Figure 6A). Together these parameters account for 35.7% of the observed species variation (27.7% by distance and inundation frequency alone). The samples cluster according to the same pattern as obtained by the DGGE analysis. Similar results were obtained for the Cyanobacteria, Archaea and Eukarya (data not shown). Incorporating pigment data into the RDA analysis revealed a similar pattern as in Figure 4, where the three DGGE clusters are linked to the different combinations of pigments (Figure 6B). Eigenvalues for axes 1 and 2 in A are 0.1611 and 0.1163, explaining 28% of total variance in the RDA and in B are 0.1795 and 0.1082 respectively explaining 29% of the total variance. The tidal zone samples correspond with the diatom related pigments (diadinoxanthin, diatoxanthin, chlorophyll-*c* and fucoxanthin), the intermediate zone samples with the cyanobacterial related pigments, especially β-carotene and zeaxanthin, and the upper zone with pigments related to the freshwater algae and cyanobacteria.

## Discussion

The diversity of microbial representatives of each of the three kingdoms of life was investigated in coastal microbial mats using molecular community fingerprinting by means of DGGE analysis. We are well aware of the limitations of DGGE analysis, such as over or underestimation due to rRNA copy number biases and chimeric sequence generation; limitations which are mainly inherent to the polymerase chain reaction [23]. Nevertheless, this technique allowed us comparing a large number of samples along a tidal transect and the microbial communities therein, especially since each sample was treated and analyzed in parallel and by the same methods.

Overall, DGGE cluster analysis of 16S and 18S rRNA gene based community fingerprints revealed three major, well-defined clusters of microbial communities along the beach transect and represent the tidal zone (TS1-TS8), the intermediate zone (TS9-TS20) and the upper zone (TS21-TS33), respectively. Each cluster consists of communities with overlapping populations of microorganisms, represented by recurring bands in the different fingerprints. Especially among the cyanobacterial fingerprints there are some bands that appear in nearly all samples. Apparently the corresponding species are not affected by the different salinities. This is in agreement with an experiment in which the salinity of a hypersaline microbial mat was varied from 35 to 85 p.p.t. and analyzed over a 5 month period [24]. These experiments revealed several cyanobacterial groups belonging to the Oscillatoriales (including *Coleofasciculus chthonoplastes* related species), that were unaffected by the salinity changes and remained stable in abundance and activity. The archaeal samples did not group in the three conserved clusters as found for the other domains of life. Repetition of the archaeal DGGE analysis starting with freshly extracted DNA from the original samples revealed identical fingerprints suggesting that the observed clustering was not caused by pipetting errors or accidentally mixing of samples. The geographical location of the samples was preserved in the three main clusters. Cluster 1 represented samples taken from the tidal zone, cluster 2 samples from the intermediate zone and cluster 3 represented the samples taken from the upper zone close to the dunes. These clusters are in agreement with previously identified microbial mat types that were characterized by microscopy and visual observation [1], [9]. Within each zone the mats appeared homogeneous with respect to a number of dominant DGGE bands causing them to cluster as a single group, but the DGGE patterns also revealed clear micro-diversity represented as unique or less frequently occurring bands within the same cluster. This is not surprising given the enormous heterogeneity in possible niches and environmental parameters in sediments as compared to the more homogenous composition of water bodies. It has been shown previously that different populations of microorganisms can be found within several millimeters depth, driven by changes in, for example, incident light, oxygen or sulfide [5], [25], [26]. Unfortunately, only a few studies have been dedicated to study the possible changes in microbial composition at the millimeter level in the horizontal plane [5], [27], [28]. Nevertheless, assuming functional redundancy in the microbial populations it is expected that the small differences observed within a fingerprint do not dramatically alter the ecophysiological properties of the mat [29]. Therefore we consider the three stations chosen in our previous study as good representatives of the different mat types, giving a valid description of the microbial community [10]. The similar clustering pattern of the micro-eukaryal, bacterial, cyanobacterial, and to a lesser extent the archaeal community fingerprints, also suggests a strong linkage of populations within these domains and groups and may hint to direct physical or syntrophic interactions between groups of organisms [30], [31].

### Characterization of the Three Zones

The samples forming the first DGGE cluster (TS01-TS08) were from the tidal zone, flanked by the low and high water mark and visible by a darker green appearance in the satellite image (Figure 1). Samples TS07 and TS09 appear to be deeper branching than the other six samples in this cluster and may already show effects of different salinities. In comparison to the higher located zones, the tidal zone reveals a lower diversity in DGGE bands of Bacteria, Cyanobacteria and Eukarya while Archaea revealed their highest diversity in this zone. The microbial composition in the tidal zone differs from the other two zones that are more similar to each other (see also Figure 6A). This is in agreement with the 454 tag-sequencing analysis, which revealed a similar clustering between the two higher located zones relative to the sample obtained from the tidal zone and also revealing the highest archaeal diversity in samples from this zone [10]. Analysis of photosynthetic pigments (Figure 4) in the tidal zone revealed a large contribution of pigments (chlorophyll-*c* and fucoxanthin and to a lesser extent diadinoxanthin and diatoxanthin) derived from diatoms [32]. In contrast, only ∼15% of the total amount of zeaxanthin and ß-carotene, characteristic for green algae and Cyanobacteria, was present in the tidal zone. This is in agreement with phospholipid-derived fatty acids (PLFA) analysis that showed that 74% of the PLFA’s extracted from the tidal zone were attributed to diatoms and <10% to Cyanobacteria [1]. These authors confirmed by microscopy the large number of diatoms in this zone with species related to *Navicula* sp., *Diploneis* sp., *Amphora* sp. and *Cylindrotheca* sp. The 454 tag-sequencing analyses also revealed a relative small contribution (<3%) of Cyanobacteria to the total bacterial richness in samples from the tidal zone. High concentrations of phaeophytin, a chlorophyll breakdown product, may be indicative for high predation on diatoms in the tidal zone [33]. Low concentrations of lutein, canthaxanthin and chlorophyll-*b* and the absence of neoxanthin suggest a minor contribution of the green lineage of algae and freshwater filamentous cyanobacterial species as was expected for a marine site. Comparison of nutrient concentrations revealed similarity between the tidal and upper zone as compared to the intermediate zone. Carbon, nitrogen and especially phosphate were highest in the tidal zone, whereas the C:N ratio in the tidal zone is only slightly lower compared to the other two zones. The average C:N value in the tidal zone of 8.4 is well above the Redfield ratio of 6.6, indicating a considerable contribution of extracellular carbon to the tidal zone ecosystem. Dijkman *et al.* (2010) [1] who sampled the same beach in 2002 found an average C:N ratio that was higher in samples taken from the tidal zone as compared to the C:N ratios found in the higher located zones. This contrast may be caused by the different periods of sampling (June 2002 in the Dijkman study versus end of August 2010 in this study) and by overall annual changes in microbial mat composition knowing to occur at these sites [9]. The prevalence of diatoms over Cyanobacteria as the dominant photosynthetic primary producers in the tidal zone could be caused by several factors. One is the higher tolerance of cyanobacteria to sulfide than diatoms [34] in the more stratified intermediate mats or the inability of mat forming Cyanobacteria to adapt to continuous inundation by salt water and mechanical stress cause by the waves. Cyanobacteria may also endure a higher stress by predation. Diatoms, on the other hand, may escape both mechanical stress and predation by their rapid vertical movement in the sediment [35]–[37].

The intermediate zone (samples TS09 through TS20) was previously identified as a *Coleofasciculus* (previously *Microcoleus*) sp. dominated mat [1], [38], [39]. In the aerial picture this zone is visible as a lighter colored zone between the tidal and upper part of the beach (Figure 1). Cluster analysis of the DGGE fingerprints revealed that this zone has more bands in common with fingerprints from the upper zone than with those of the tidal zone. The intermediate zone is characterized by the highest average C:N ratio, suggesting that a large amount of EPS is exuded in the environment. This is in agreement with visual observations of the mats that have a mucous constitution. The average concentrations of total carbon, nitrogen and phosphate, normalized for chlorophyll *a*, are significant lower compared to the other two zones. This is probably caused by the fact that the mats from the intermediate zone have the highest amount of standing stock biomass. The strong self-shadowing in the mats may cause an accumulation of chlorophyll (giving these mats an almost black appearance) and hence lower values for C, N and P after normalization.

Pigment signatures show that, compared to the tidal zone, Cyanobacteria are more abundant in the intermediate zone with higher concentrations of zeaxanthin and beta-carotene while diatoms are less abundant. The higher contribution of Cyanobacteria in this zone is in agreement with PLFA analysis [1] and 454 tag-sequencing with >20% Cyanobacteria amongst the total bacterial community [10]. Low concentrations of lutein, canthaxanthin and chlorophyll-*b* suggest a minor contribution of the green lineage of algae and of freshwater filamentous cyanobacterial species to the intermediate zone and are nearly absent in the tidal zone. Total pigment and nutrient concentrations in the intermediate zone varied widely. Sample TS09 at the border of the tidal and intermediate zone revealed a more than 2 fold higher biomass in terms of total carbon and pigment concentration as compared to the other samples. These fluctuations, which to a lesser extent are also found in the upper zone, may reflect the patchiness observed within the microbial mats. Mats of different thickness and age can be found that are formed during the annual cycle of microbial mat formation. Mats at some locations are completely destroyed by the forces of nature leaving bare sand on which new mats may develop, whereas other mats develop on top of older mats similar to the formation of stromatolites [40]. Despite large fluctuations in nutrient and pigment concentrations, the dominant community structure as determined by DGGE analysis remains similar causing them to group in a single cluster. The diversity within the cyanobacterial community significantly increased in the intermediate zone as compared to the tidal zone. Species related to *Coleofasciculus* (*Microcoleus*) sp., *Lyngbya* sp., *Pleurocapsa* sp., *Symploca* sp., *Synechococcus* sp., *Leptolyngbya* sp., *Microcystis* sp., *Nostoc* sp. and species belonging to the cluster of heterocystous cyanobacteria (GpI) that are nearly absent from the tidal zone are abundant in the intermediate and upper zones [10]. These species are probably better adapted to the brackish environment of the intermediate zone rather than to the continuous inundated marine conditions of the tidal zone. In contrast to the total bacterial- and eukaryote diversity, the archaeal diversity decreased relative to the tidal zone. A similar observation was made by 454 analysis where the decrease in diversity of the dominant archaeal population was attributed to an explosive growth of halophilic Archaea during the hot summer months which masked the underlying rare diversity [10].

The upper zone (TS21 to TS33) is largely overgrown by diverse salt marsh vegetation (Figure 1). The microbial communities in this zone group as a separate cluster but are more similar to the intermediate zone than to the tidal zone, in agreement with results obtained by pyro-sequencing analysis [10]. The significant lower diversity indices obtained for the archaeal community in the upper zone is also in agreement with that study, where closely related groups of methanogenic Archaea dominated the sample obtained from the upper zone. Despite clear overlap in community composition between the upper and intermediate zone this overlap is not so obvious from the pigment analysis. All pigments, with the exception of diatoxanthin and beta-carotene, were present at a higher content in the upper zone compared to the intermediate zone. This shows that also in the upper zone diatoms and Cyanobacteria are abundantly present, which is in agreement with microscopy observations. The upper zone contained the highest contents of zeaxanthin, lutein, canthaxanthin, chlorophyll-*b* and neoxanthin. This suggests a higher contribution of green lineage of algae (e.g. Chlorophyta, Prasinophyta, Euglenophyta), cyanobacterial species of the order Prochlorales and of freshwater filamentous cyanobacterial species (*Prochlorothrix*) [32] and is in agreement with a higher input of freshwater in this part of the beach.

RDA analysis confirmed the sharp separation between the tidal and intermediate zone communities and the more gradual change from the intermediate to the upper zone (Figure 6). Checking this to the theory of the distribution of vegetation along gradients, we conclude that the two contrasting theories (gradual change [13] versus tight linkage [14]) both apply for the observed microbial diversity in the microbial mats studied here. The boundary between the tidal and intermediate zones appears to form a *limes convergens* or a sharp boundary for all groups of microorganisms, whereas the boundary between the intermediate and the upper zone represents a *limes divergens* [15], where several species (DGGE bands) co-occur. However, such strict boundaries are hard to envision from a microbial community and environmental gradient point of view. Even though the boundary between the tidal and intermediate zone based on DGGE analysis appears to be sharp, it is subject to continuous short term changes. The position of the high and low water marks shifts on a daily basis depending on the lunar cycle and, hence, the frequency of daily inundation and exposure to the concomitant environmental factors varies largely in time and space. We approximated the boundary between the tidal and intermediate zone by visual inspection of the extant high water mark and on hindsight by analyzing the DGGE results. The overlap of the intermediate and upper zones is also subject to change because the vegetated zone is continuously expanding towards the sea, decreasing the area of the intermediate zone. Most likely a climax microbial community has not yet established in the intermediate to upper zone. The distinct community structures are associated with the distance from the low water mark and concomitant beach elevation. Parameters that are linked to this transect are frequency of inundation and exposure to rainfall both affecting salinity, water content and desiccation stress. Similar studies are rare and limited to extreme environments, such as hypersaline ponds or hot springs or restricted to a single group of microorganisms [41]. In a DGGE based study of microbial mats in a hypersaline coastal lagoon in Guerrero Negro, Baja California Sur, Mexico, samples were taken at large intervals over a 1 km stretch and included a tidal zone [42]. These authors concluded that of all the environmental factors co-varying with intertidal height, desiccation frequency was the most dominant. Salinity changes as shaping force were excluded in that study because rainfall in the area was rare and resulted in minimal variations in salinity over the sampled transects. However, other studies revealed effects of salinity on community structure in hypersaline mats that were exposed to different salinities [43], [44]. It is difficult to compare hypersaline mats with the coastal mats studied by us. More close to this is a study along a natural salinity gradient in a 25 km long estuary where bacterial diversity was assessed in surface water using DGGE [45]. Similar as in our microbial mats, this study also showed that the bacterial community composition changed along the estuarine gradient but it failed to point out the actual driver of the observed diversity.

In conclusion, community fingerprint analysis of a transect of the North Sea beach of Schiermonnikoog confirms the existence of the three major zones each consisting of their own type of microbial mat and specific composition as determined previously [10]. In addition to the major DGGE clusters, small differences in community composition exist between the independent samples and underline the high microbial diversity found in coastal microbial mats. Furthermore, there appears to be a tight coupling (similar clustering of community fingerprints) between the different domains of life that co-occupy the same mat type, especially in the tidal zone. In the intermediate and upper zone this coupling is weaker especially for the Archaea. This is probably the result of the large fluctuations in environmental conditions in the intermediate and upper zones due to infrequent inundation and variable periods of rain. Although it is conceivable that salinity is the mayor driver of microbial community composition in these coastal microbial mats, there are a number of other co-varying factors which could also drive the observed changes in community composition. Identifying these dominant driver(s) requires quantitative experimental studies of microbial communities exposed to different environmental conditions.
